# Smart diabetic foot ulcer scoring system

**DOI:** 10.1038/s41598-024-62076-1

**Published:** 2024-05-21

**Authors:** Zheng Wang, Xinyu Tan, Yang Xue, Chen Xiao, Kejuan Yue, Kaibin Lin, Chong Wang, Qiuhong Zhou, Jianglin Zhang

**Affiliations:** 1 School of Computer Science, Hunan First Normal University, Changsha, 410205 China; 2 Department of Dermatology, Shenzhen People’s Hospital, The Second Clinical Medical College, Jinan University, The First Affiliated Hospital, Southern University of Science and Technology, Shenzhen, 518020 Guangdong China; 3 Department of Clinical Nursing, Xiangya Hospital, Central South University, Changsha, 410008 China; 4 Candidate Branch of National Clinical Research Center for Skin Diseases, Shenzhen, 518020 Guangdong China; 5 Department of Geriatrics, Shenzhen People’s Hospital, The Second Clinical Medical College, Jinan University, The First Affiliated Hospital, Southern University of Science and Technology, Shenzhen, 518020 Guangdong China; 6 Foot Prevention and Treatment Center, Xiangya Hospital, Central South University, Changsha, 410008 China

**Keywords:** Diabetic foot ulcers, Deep learning, Biomedical image analysis, Transfer learning, Diseases, Health care

## Abstract

Current assessment methods for diabetic foot ulcers (DFUs) lack objectivity and consistency, posing a significant risk to diabetes patients, including the potential for amputations, highlighting the urgent need for improved diagnostic tools and care standards in the field. To address this issue, the objective of this study was to develop and evaluate the Smart Diabetic Foot Ulcer Scoring System, ScoreDFUNet, which incorporates artificial intelligence (AI) and image analysis techniques, aiming to enhance the precision and consistency of diabetic foot ulcer assessment. ScoreDFUNet demonstrates precise categorization of DFU images into “ulcer,” “infection,” “normal,” and “gangrene” areas, achieving a noteworthy accuracy rate of 95.34% on the test set, with elevated levels of precision, recall, and F1 scores. Comparative evaluations with dermatologists affirm that our algorithm consistently surpasses the performance of junior and mid-level dermatologists, closely matching the assessments of senior dermatologists, and rigorous analyses including Bland–Altman plots and significance testing validate the robustness and reliability of our algorithm. This innovative AI system presents a valuable tool for healthcare professionals and can significantly improve the care standards in the field of diabetic foot ulcer assessment.

## Introduction

Diabetes mellitus (DM) is a globally prevalent, chronic metabolic disorder characterized by persistent hyperglycemia, with projections indicating that its global prevalence will affect around 700 million adults by 2045^1,2^. DM poses a significant health burden due to its association with severe complications, including myocardial infarction, stroke, blindness, renal failure, and the risk of lower limb amputations, substantially impacting both mortality and quality of life^3^. Notably, diabetic foot ulcers (DFUs) are a substantial and prevalent concern, afflicting a considerable percentage of diabetic patients, with reported prevalence ranging from 19 to 34%^4^. The consequences of inadequate healthcare for DFUs can be dire, often resulting in lower limb amputations, with over a million individuals worldwide undergoing such procedures annually. Managing high-risk foot patients requires regular medical assessments, medication adherence, and vigilant self-care, imposing substantial economic burdens, particularly in developing nations^5^.

Key risk factors for diabetic foot infections encompass neuropathy, peripheral vascular disease, and suboptimal glycemic control, collectively leading to reduced sensory perception, compromised perspiration, foot deformities, and circulatory impairments^6–8^. This intricate interplay can exacerbate skin complications and microbial infections, impeding the healing of ulcers. Recent research, including the application of deep learning techniques, underscores the critical importance of early diagnosis and comprehensive healthcare for patients with these risk factors, highlighting the pressing need for advanced methodologies in DFU diagnosis and management^9–12^.

Traditional methods for assessing the severity of diabetic foot ulcers (DFUs) are known to be time-consuming, labor-intensive, and subject to variations among physicians, hindering precise patient monitoring. In response to this challenge, our study presents an innovative automated scoring system designed to objectively evaluate the severity of DFUs by identifying ulceration, infection, and gangrene features in diabetic foot ulcer images. This system provides dependable assessments that can inform personalized treatment and healthcare plans, aligning with previous research demonstrating the potential of machine learning algorithms in DFU recognition. Prior studies have explored various aspects of early diagnosis, wound segmentation, classification, and tailored approaches in DFU management^13–24^.

Built upon deep learning, we introduce the Smart Diabetic Foot Ulcer Scoring System (ScoreDFUNet) to assess diabetic foot ulcers with precision. ScoreDFUNet facilitates the accurate quantification of diabetic foot ulcer severity, encompassing lesion size and infection status, and offers predictive diagnostic scores. These scores can assist dermatologists in planning and implementing appropriate treatment strategies. To further validate the feasibility and effectiveness of ScoreDFUNet, we investigated its relationship with expert dermatologists' assessments. This study represents a significant advancement in the domain of diabetic foot ulcer assessment and signifies the potential of AI-driven solutions to enhance patient care in this critical area of healthcare.

## Methods

### Datasets and ethical approval

This study was conducted with the approval of the Shenzhen People's Hospital Scientific Research Ethics Committee (approval number: LL-KY-2023120-01). Informed consent was obtained from the patients, ensuring the privacy and confidentiality of their information. All methods were carried out in accordance with relevant guidelines and regulations.

The image dataset comprised 1426 high-definition diabetic foot ulcer images, with 603 obtained from the Diabetic Foot Ulcer Challenge (DFUC) 2020^25^ online dataset and 823 provided by the Endocrinology Department at Xiangya Hospital. Patients' ages ranged from 5 to 72 years, with an average age of 38.75 years, and the gender distribution was 40% men and 60% women. Data collection at Xiangya Hospital occurred from April 2015 to December 2020, using an iPad positioned approximately 20 cm from the ulcers, with default camera settings. To augment the dataset, we incorporated 518 images from Kaggle^26^, categorized into normal and ulcerated groups, with 440 as normal and 78 depicting diabetic foot ulcers. We used the Foot Ulcer Segmentation Challenge 2021 dataset^27^ for our external test set. Out of 1210 images, 923 showed full feet. From these, we chose 595 images that clearly displayed only the foot, without the lower leg, to enhance model training efficiency and assess its real-world applicability. The detailed information depicted in Table 1.

**Table 1:** Distribution of class instances and datasets.

	Train	Validation	Internal test	Total	External test
Class instances					
Gangrene	374 (27.7%)	108 (27.8%)	61 (29.7%)	543	100 (17%)
Normal	302 (22.4%)	89 (22.9%)	49 (23.9%)	440	0 (0%)
Ulcer	344 (25.5%)	97 (25%)	48 (23.4%)	489	346 (57%)
Infection	332 (24.5%)	94 (24.2%)	46 (22.9%)	472	149 (26%)
Datasets					
DFUC 2020	422 (31.2%)	119 (30.7%)	62 (30.4%)	603	–
Xiangya hospital	594 (43.9%)	164 (42.3%)	65 (31.9%)	823	–
Kaggle	336 (24.9%)	105 (27.1%)	77 (37.7%)	518	–
Total	1352 (70%)	388 (20%)	204 (10%)	1944	595

### Score net of risk stratification for DFU

To assess the dataset, we engaged three dermatologists with varying levels of experience, including a junior physician (3+ years), a mid-level physician (5+ years), and a senior physician (10+ years).

**Figure 1:**
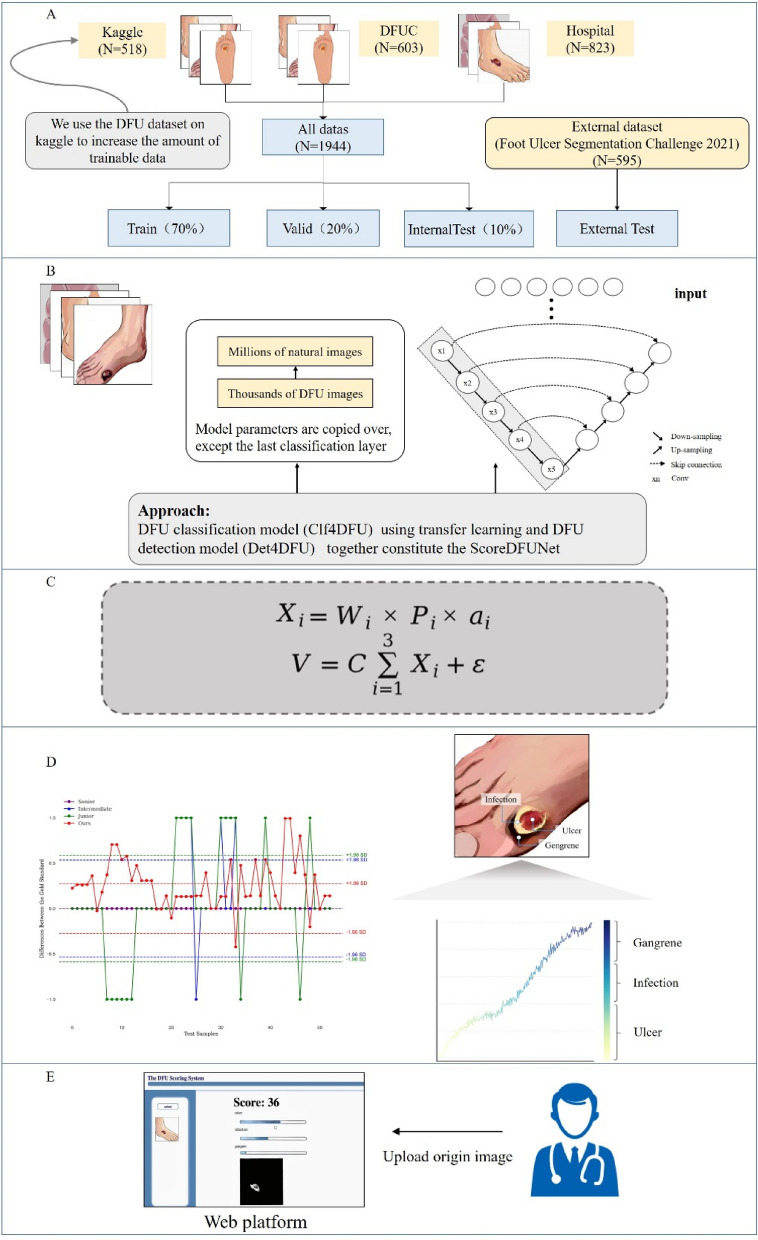
Framework for diabetic foot ulcer assessment. (**A**) Internal phase were split into three sets with a ratio of 7:2:1, comprising the training, validation and test sets. Foot Ulcer Segmentation Challenge 2021 data is for external test. (**B**) Two models were employed, one for diabetic foot ulcer stratification and the other for lesion identification and segmentation. (**C**) The scoring system for diabetic foot ulcers is founded on mathematical principles. (**D**) Algorithmic scores from the test dataset were consistently compared with physician-assigned scores to identify specific lesion features. (**E**) Dermatologists uploaded original diabetic foot ulcer photos into the intelligent scoring platform, which generated corresponding scores.

In the model training phase (as illustrated in Fig. 1), we employed the U-Net architecture^28,29^ for the multi-category segmentation task in diabetic foot ulcer analysis, allowing precise capture of distinct features and enhancing segmentation accuracy. The ground truth data was carefully annotated by physicians to ensure accuracy based on Wagner's classification system^12^. Simultaneously, we selected ResNet50^30–34^ as the foundational network for classifying lesion features of diabetic foot ulcers. Blanco et al.^30^ presented QTDU, a new method based on ResNet50 to accurately identify and measure tissues in dermatological ulcers. Jaganathan et al.^34^ investigated how taken with mobile photographs, alongside ResNet50, can improve the assessment and treatment of chronic wounds by accurately classifying and measuring them. Utilizing pre-trained model weights from ImageNet, we implemented transfer learning, accelerating model convergence and significantly improving overall performance.

By combining the outputs of the U-Net and ResNet50 models, we developed an innovative algorithm for the objective assessment of diabetic foot ulcer severity. This algorithm offers dermatologists a reliable scoring method for in-depth analysis of diabetic foot ulcer images, accessible through a user-friendly online platform. This platform streamlines the process for dermatologists, allowing them to upload patient photos of diabetic foot ulcers and access comprehensive feedback on a dedicated web page related to the rating.

For the detection of skin lesions in diabetic foot ulcers, our innovative approach, termed Det4DFU, represents a departure from previous methods that primarily focused on lesion localization^35^. Utilizing the U-Net architecture, our two-stage segmentation process significantly enhances accuracy and clinical relevance. In the initial stage, we prioritize binary segmentation to precisely distinguish between normal and diseased regions, providing a robust foundation for subsequent tasks. In the second stage of our analysis, we employ advanced multi-class segmentation techniques to refine and categorize skin lesion areas into six specific categories. These categories include Ulcer1 (superficial ulcers), Ulcer2 (deep ulcers), Infection1 (soft tissue infections), Infection2 (deep abscesses or osteomyelitis), Gangrene1 (localized gangrene), and Gangrene2 (whole-foot gangrene). These categories^12^ reflect the varying severity of diabetic foot skin lesions, quantifying both the depth and extent of lesion features. This detailed classification aids in maximizing the use of image information for scoring purposes, enhancing the precision and utility of our scoring system.

**Figure 2:**
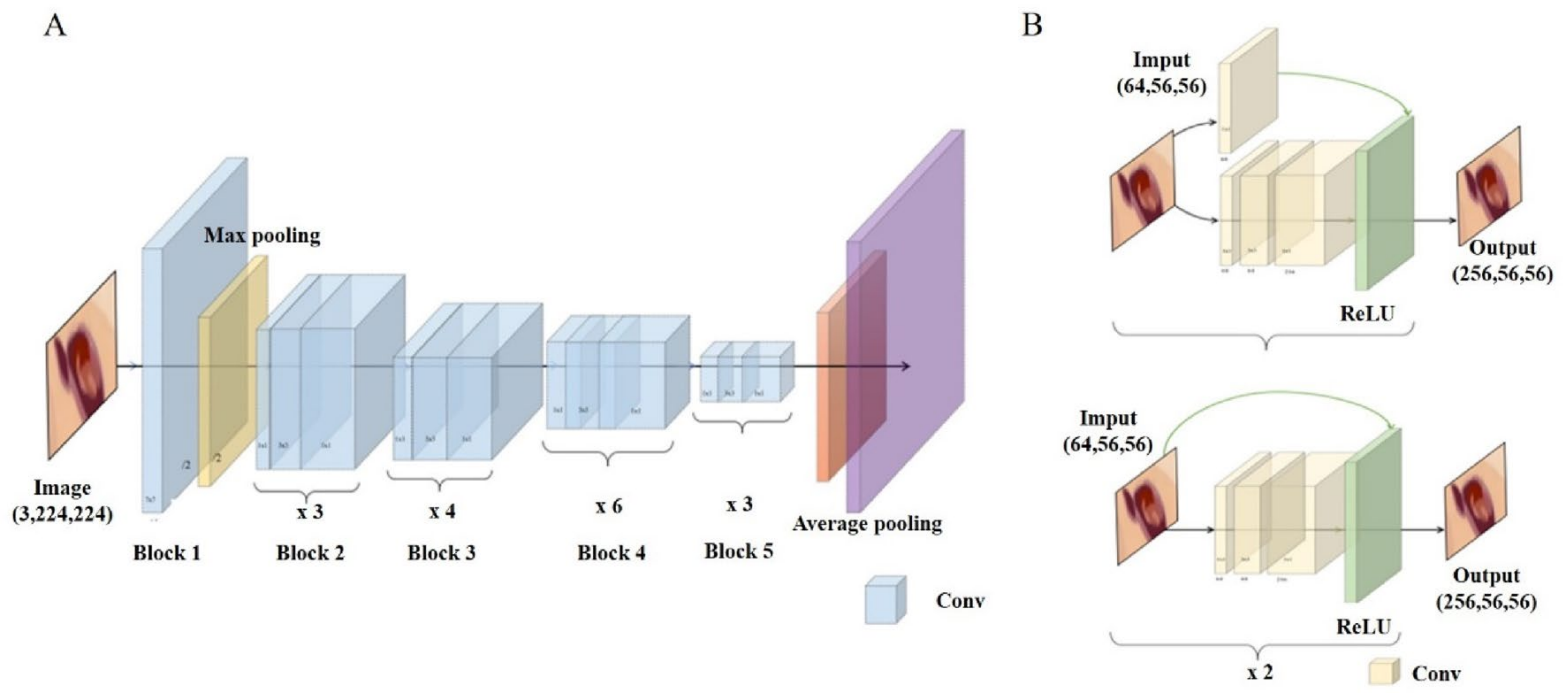
Architecture of stratification model. Our stratification model is based on the ResNet50 architecture, specifically designed for classifying severity categories. ResNet50 utilizes residual blocks to enhance information flow across its layers and consists of convolutional layers, residual blocks, global average pooling, and fully connected layers.

For the stratification of diabetic foot ulcer severity, our model, Clf4DFU, leverages the ResNet50 neural network architecture as the backbone with innovative residual learning, as illustrated in Fig. 2. This approach effectively mitigates vanishing gradient issues. Comprising convolutional layers, residual blocks, global average pooling, and customized fully connected layers, ResNet50 achieves precise predictions. The fully connected layer's output is redefined to represent four severity categories: "normal," "infection," "ulcer," and "necrosis." Training is expedited by leveraging ResNet50's pre-trained parameters from ImageNet, resulting in enhanced performance and efficiency compared to training from scratch.

### Transfer learning

In our approach, transfer learning plays a fundamental role, drawing from well-established machine learning principles. Through the utilization of transfer learning, we harnessed pre-trained model weights originally designed for more comprehensive tasks and applied them to the specific challenges of classifying and segmenting diabetic foot ulcer photos^36–38^. This concept aligns with the idea that models equipped with features learned from extensive and diverse datasets, such as ImageNet, can excel in tasks with limited labeled data. Such an approach enriches our model's understanding of diabetic foot ulcer photos, enabling it to capture essential features crucial for stratification and segmentation. Importantly, transfer learning not only enhances our model's performance but also significantly reduces training time, ultimately increasing the efficiency and precision of the severity assessment method for diabetic foot ulcers.

### Evaluation metrics

In our study, we employed a comprehensive set of evaluation metrics to rigorously evaluate the accuracy of our stratification and segmentation results. These metrics played a dual role by quantifying our model's performance and establishing a robust theoretical basis for interpreting our experimental outcomes. To thoroughly assess the predictive capabilities of our segmentation and stratification algorithm models, we incorporated the following essential performance metrics:

#### Accuracy

1 $$ {\text{Eva}}_{Accuracy} = \frac{TP + TN}{{TP + FP + TN + FN}} $$

#### Precision

2 $$ Eva_{Precision} = \frac{TP}{{TP + FP}} $$

#### Recall

3 $$ Eva_{Recall} = \frac{TP}{{TP + FN}} $$

#### F1-score (F1)

4 $$ Eva_{F1} = 2 \times \frac{Precision \times Recall}{{Precision + Recall}} $$

#### Dice coefficient

5 $$ Eva_{Dice} = \frac{2 \times TP}{{FP + 2 \times TP + FN}} $$

#### Mean IoU (MIoU)

6 $$ Eva_{MIoU} = \frac{1}{k + 1} \mathop \sum \limits_{i = 0}^{k} \frac{TP}{{FN + FP + TP}} $$

### Scoring strategy

To enhance the precision of assessing diabetic foot ulcer photos and reduce potential variations in clinicians' judgments, we introduced an innovative scoring methodology. This approach involves the utilization of specific area measurements, denoted as S_i_, to represent the distinct characteristics of infection, ulcer, and gangrene within diabetic foot ulcer images. The corresponding feature ratios for each characteristic are defined as follows:

7 $$ a_{i} = \frac{{S_{i} }}{{\mathop \sum \nolimits_{i = 1}^{3} S_{i} }} $$

where $a_{i}$ denotes the proportion of the specific feature's area in relation to the total area encompassing all three characteristics detected within the diabetic foot ulcer image.

These three features exhibit distinct behaviors at various severity levels. Therefore, we categorize them into two distinct degrees, denoted as S_i1_ and S_i2_, to represent each feature. We employ X_i_ to express the feature score for each characteristic, as follows:

8 $$ X_{i} = W_{i} \times P_{i} \left( {\frac{{(W_{i1} \times S_{i1} + W_{i2} \times S_{i2} )}}{{\mathop \sum \nolimits_{i = 1}^{3} S_{i} }}} \right) $$

where the feature ratio for each characteristic is established by standardizing the weights W_i_ assigned to different features, as well as the weights W_i1_ and W_i2_ corresponding to features at different levels.

The confidence in each feature is determined by calculating the category probability output P_i_ obtained from the stratification model. This method considers feature performance, thereby increasing the level of evidence incorporated into the computation of feature scores. The total feature scores are then computed using the variable V and are expressed as follows:

9 $$ V = C\mathop \sum \limits_{i = 1}^{3} W_{i} \times P_{i} \left( {\frac{{(W_{i1} \times S_{i1} + W_{i2} \times S_{i2} )}}{{\mathop \sum \nolimits_{i = 1}^{3} S_{i} }}} \right) + \varepsilon $$

where $C$ is employed to standardize the range of evaluation scores. In situations where score deviations are observed, we have introduced a special ε to make adjustments to the total score, ensuring a more reasonable and objective scoring process. Algorithm 1 offers more details on our suggested functions.

**Figure 3:**
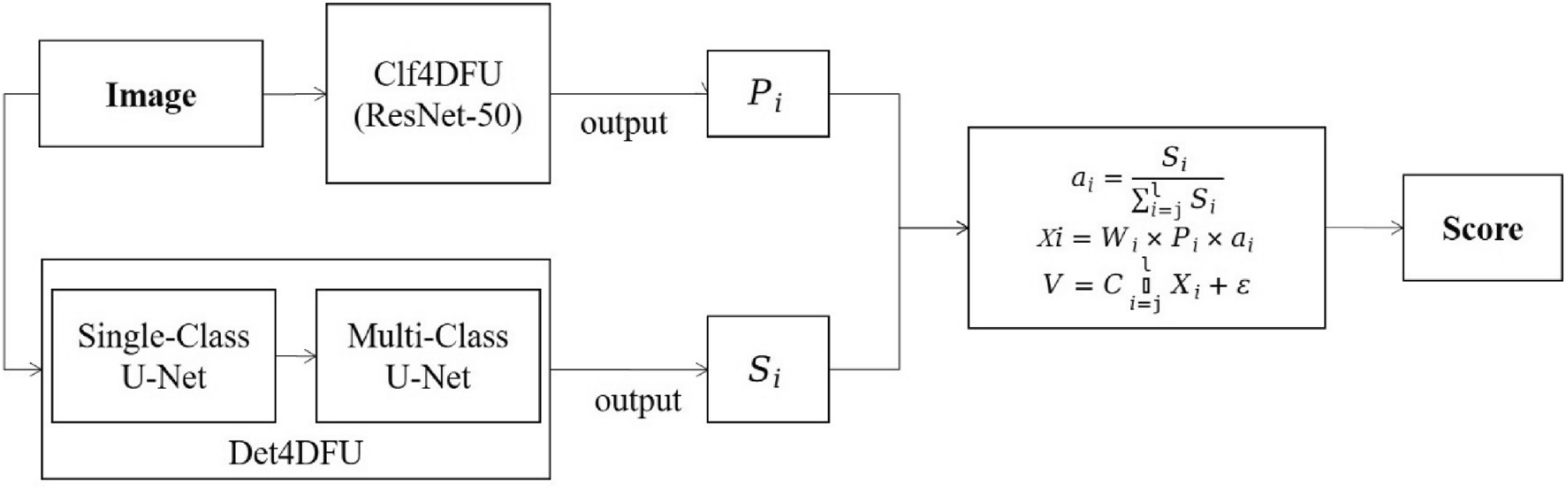
Block diagram of workflow.

Our scoring strategy is depicted in Fig. 3, where the target image undergoes dual analysis through classification and segmentation models. Initially, the classification model generates probability lists for each lesion category. Concurrently, the segmentation model isolates the lesion using a single-class approach, followed by a multi-class model to classify the lesions into six distinct categories. The areas of these segmented lesions are then calculated to ascertain their extent. Ultimately, the data from both models are integrated, applying our specialized scoring formula to derive the final score for the image.

**Figure:**
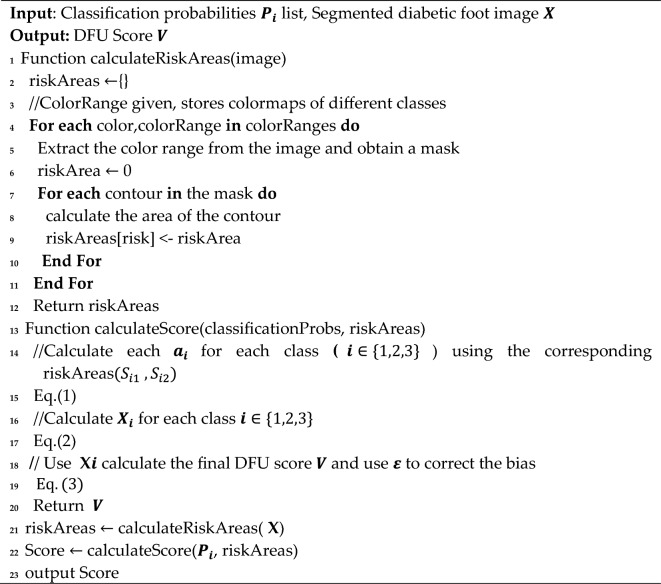
Algorithm 1 Scoring Strategy for Stratifying Diabetic Foot Ulcers

## Results

### ScoreDFUNet model results

**Figure 4:**
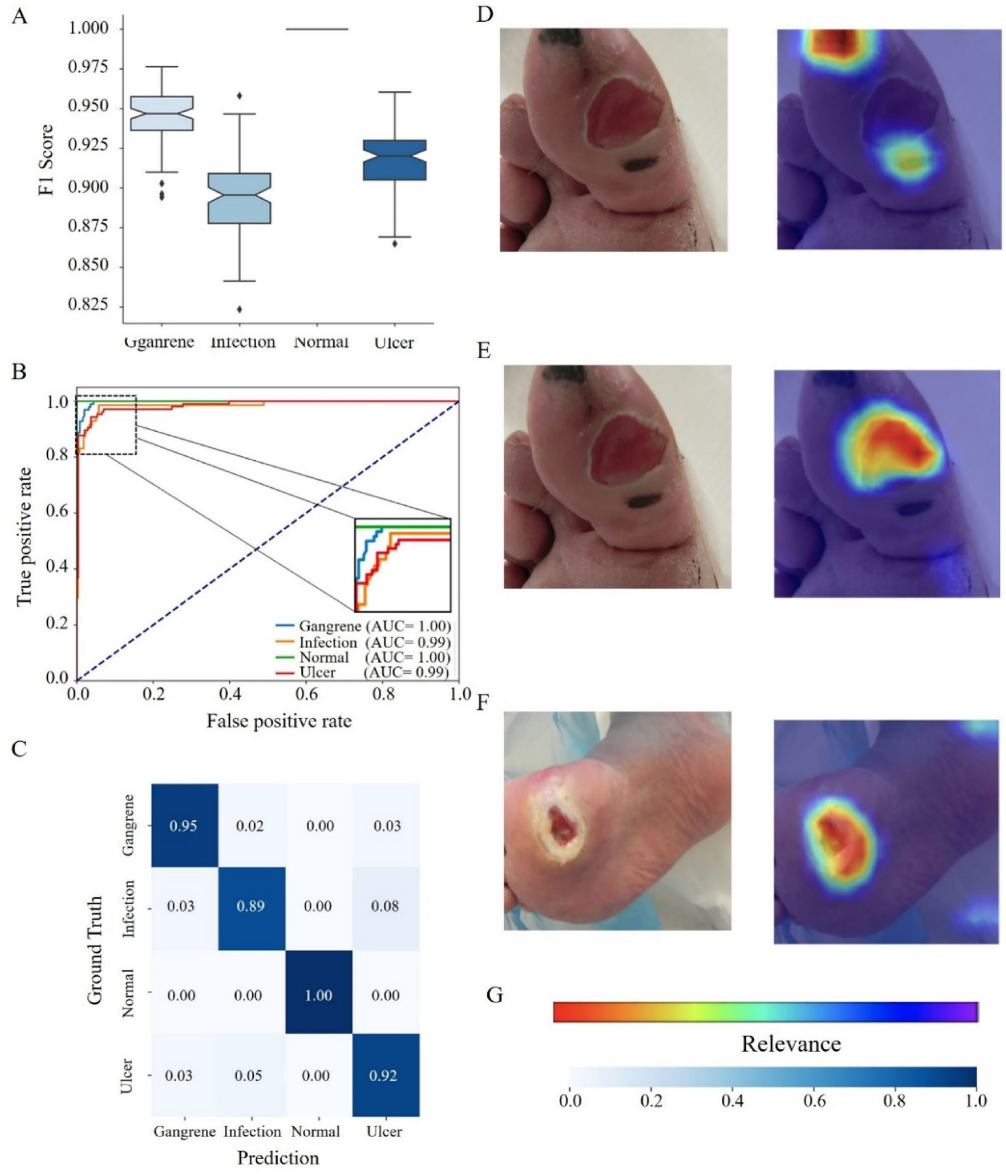
Performance analysis of ScoreDFUNet. (**A**) F1-score Performance: Illustrates the F1-score performance of the Clf4DFU model, showcasing its ability to achieve a balanced trade-off between precision and recall in diabetic foot ulcer classification. (**B**) ROC Curve: Presents the receiver operating characteristic (ROC) curve, highlighting the model's effectiveness in distinguishing between different lesion categories. (**C**) Confusion Matrix: Provides a comprehensive breakdown of the model's stratification results, facilitating a detailed assessment of its accuracy and potential areas for improvement. (**D–F**) Feature Influence Analysis: Offers both qualitative and quantitative saliency analysis, emphasizing the significance of various diabetic foot ulcer features in influencing the model's predictions. (**G**) Relevance Colorbar.

In this section, we present the outcomes of our deep learning model, ScoreDFUNet, which utilizes the ResNet50 architecture to accurately stratify diabetic foot ulcer photos. We systematically categorized our dataset into four essential groups: "ulcer," "infection," "normal," and "gangrene," representing key aspects of diabetic foot ulcer pathology. Our model excels in extracting vital information from these images by analyzing the probability distribution of these categories. To enhance the interpretability of our model's performance (as illustrated in Fig. 4), we employed Grad-CAM^39^, a visual representation tool, to highlight relevant image features. Rigorous validation on a separate dataset demonstrated the model's impressive accuracy of 95.34%. Furthermore, our model exhibited strong performance metrics, including high precision, recall, and F1 scores across the categorization categories. These results underscore the efficacy of ScoreDFUNet in advancing the field of diabetic foot ulcer image analysis, potentially improving diagnostic accuracy and patient care.

**Figure 5:**
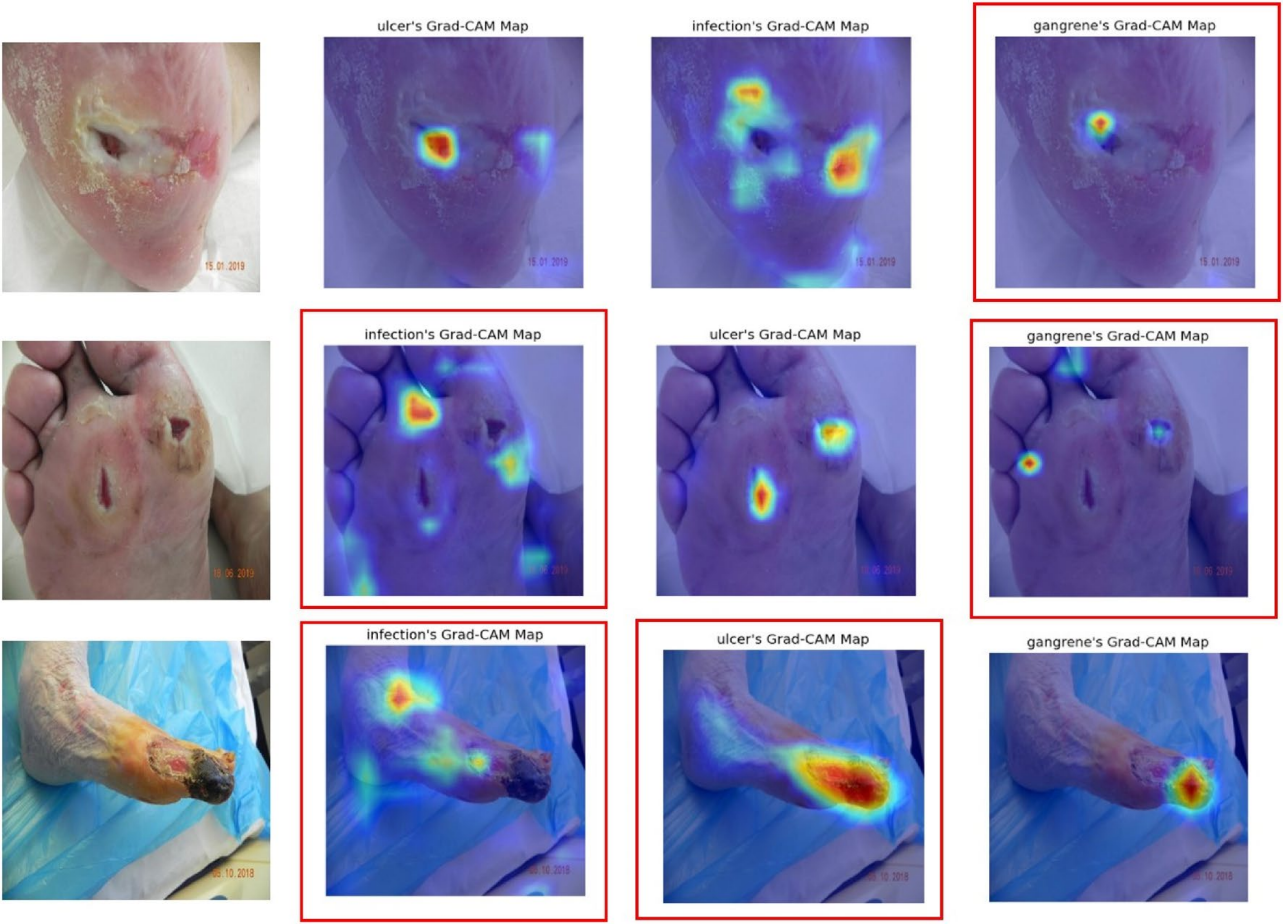
CAM examples with low consistency. CAM highlighted by red boxes depict instances of low consistency between human and machine assessments.

We conducted an in-depth examination of a subset of CAM images, particularly those where the evaluations by human experts and our model did not align, as shown in the Fig. 5. Our analysis suggests that the model's misidentification of gangrene could be attributed to its misrecognition of areas with color resemblances, such as dark skin folds or shadows in the wound. Challenges in infection identification also seemed to be affected by color similarity. While the model generally performed well with ulcers, it sometimes confused gangrene for ulcers, potentially due to certain images in the dataset displaying characteristics of both conditions, which may have led to less accurate ulcer CAM images.

### Comparative analysis with dermatologists

**Figure 6:**
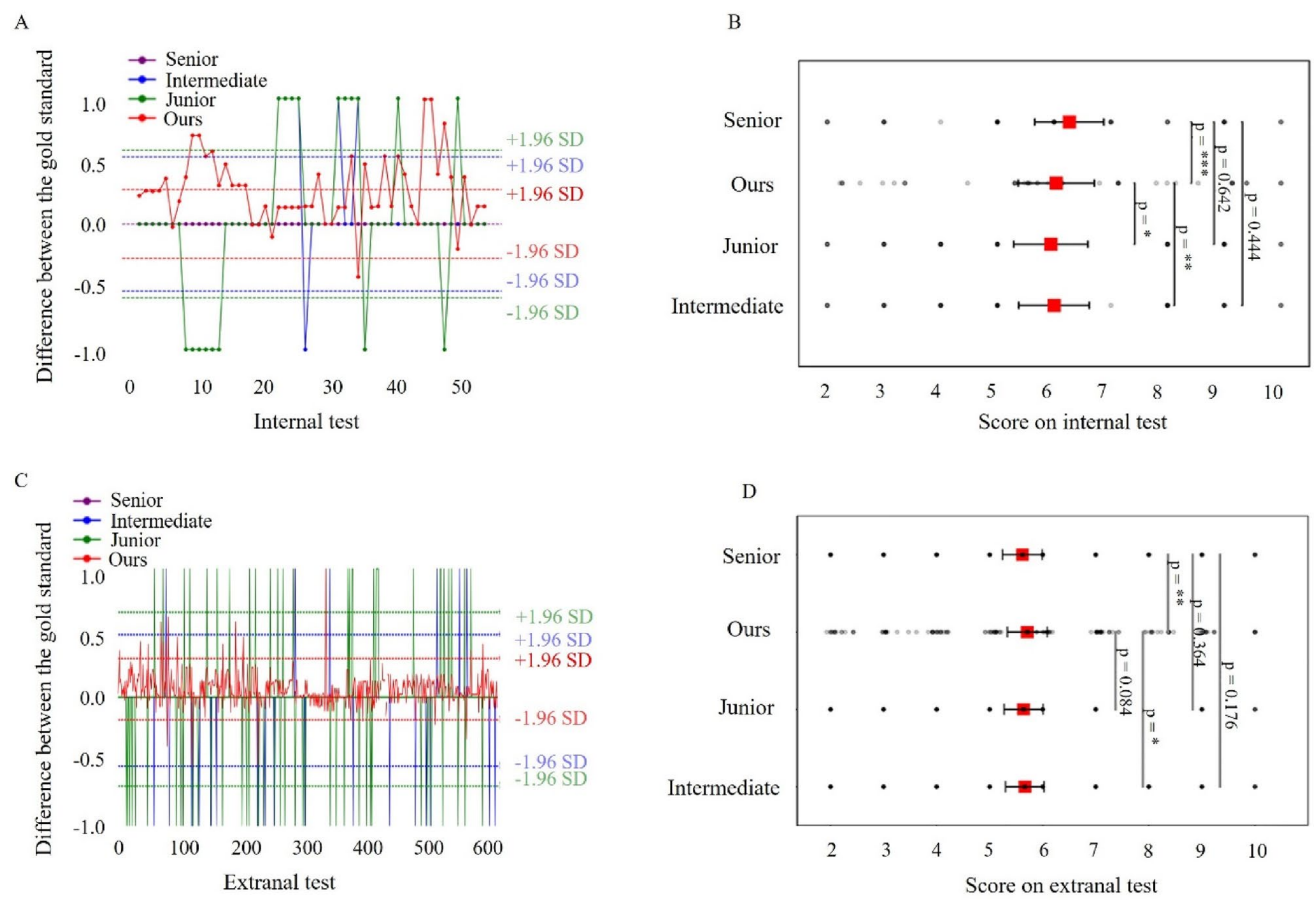
Comparative analysis: algorithm versus clinical scores. (**A**) Illustrates the differences in scores on the internal test. (**B**) Analyzes the *p* values derived from the internal test. (**C**) Displays the score differences on the external test set. (**D**) Analyzes the p-values derived from the external test. *, **, and *** to denote significance levels of test below 0.05, 0.01, and 0.001, respectively.

Our research included a meticulous comparison of our algorithm's performance with clinical scores assigned by dermatologists of varying expertise levels, with senior dermatologists' assessments serving as the reference standard (as illustrated in Fig. 6). Our algorithm consistently outperformed mid-level and junior dermatologists, closely aligning with senior dermatologists' assessments. This high level of agreement significantly enhances the accuracy of diabetic foot ulcer severity estimation. Notably, our algorithm consistently yielded slightly higher scores than clinical scores, highlighting its proficiency in stratifying the severity of diabetic foot ulcers.

We addressed the inherent subjectivity in clinical scoring by introducing objective, quantitative features, enhancing the objectivity of patient evaluations and treatment decisions. Our comparative analysis utilized Bland–Altman plots and comprehensive statistical analysis to illustrate the differences and assess the significance of the differences between our algorithm's scores and clinical ratings. This research emphasizes the superiority of our deep learning-based scoring approach, highlighting its robust performance and potential to reshape clinical diagnosis and healthcare for individuals with diabetic foot ulcers.

### Disease assessment framework results

**Figure 7:**
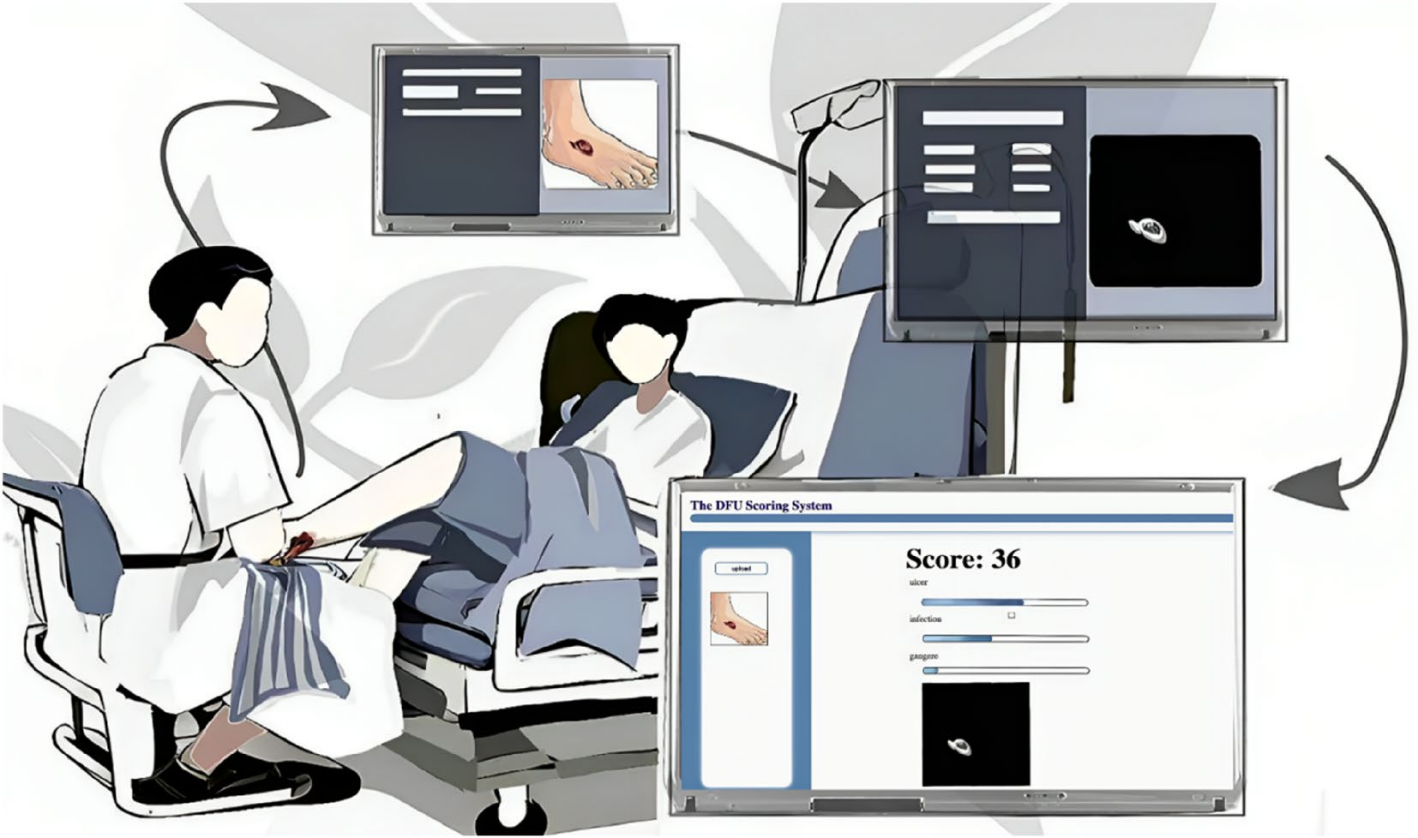
Diabetic foot ulcer assessment framework. This framework symbolizes the integration of advanced technology with clinical practice, offering a valuable tool to enhance the efficiency and precision of diabetic foot ulcer disease assessment.

Our proposed approach was successfully implemented in real clinical settings through a customized web platform designed for dermatologists. This platform seamlessly integrates our methodology, providing an objective stratification of diabetic foot ulcer photos (as illustrated in Fig. 7). This transformation offers a more reliable and quantifiable foundation for severity assessment, thereby enhancing the precision of clinical evaluations^40–43^.

Through our platform, dermatologists gain efficient access to severity assessments of diabetic foot ulcer patients, enabling a deeper understanding and more accurate assessment of the patient's healthcare needs. Furthermore, the integration of our scoring system holds the potential for optimizing treatment planning. With precise severity stratifications provided by our approach, clinicians can tailor highly personalized treatments, enhancing treatment effectiveness and alignment with the specific needs dictated by the patient's condition.

In contrast to traditional manual diagnosis by medical professionals, our scoring platform provides significant advantages in terms of speed, convenience, and consistency. Our method produces precise scoring results in just 15 s, highlighting the efficiency and cost-effectiveness of our healthcare solution. Moreover, our approach consistently correlates with the assessments conducted by expert physicians, ensuring dependable and high-quality results.

## Conclusions

In this study, we have presented a comprehensive model for the assessment of diabetic foot ulcers (DFUs) that encompasses lesion detection, segmentation, stratification, and severity scoring based on DFU photographs. Our model closely aligns with the assessments of senior dermatologists, providing a reliable and objective method for evaluating DFU severity, achieving an impressive detection accuracy of 95.34% across lesion categories. The practical utility of this model represents a substantial step toward enhancing DFU healthcare in clinical settings. Through a user-friendly web application, healthcare professionals can efficiently upload patient photos in real-time and promptly receive algorithm-generated scores. This seamless integration of technology bridges the gap between data-driven insights and hands-on patient care, positioning it as a valuable asset for real-world scenarios and promising to revolutionize DFU management.

While this study has achieved significant progress, there are considerations for further refinement. Variations in the angles, distances, and partial views in the photographs used may limit the assessment of the overall foot condition, potentially leading to scoring discrepancies in larger-scale conditions. Acknowledging this limitation, future endeavors will prioritize standardizing the collection of clinical photos to create a more comprehensive and precise scoring model. This ongoing commitment to model enhancement underscores our dedication to advancing the field of diabetic foot ulcer healthcare and ultimately improving patient outcomes.

In summary, our study presents a promising model for diabetic foot ulcer (DFU) assessment, achieving an impressive 95.34% detection accuracy across lesion categories and closely aligning with senior dermatologists' assessments. The practical application of this model through a user-friendly web platform holds the potential to enhance DFU healthcare in clinical settings, bridging the gap between data-driven insights and hands-on patient care. Despite some variations in photograph angles and views, future refinements in standardizing image collection aim to further improve the model's accuracy. This work underscores our commitment to advancing DFU healthcare and ultimately benefiting patients.

## Data Availability

The datasets generated and analyzed during this study are available in the Kaggle repository at https://www.kaggle.com/datasets/laithjj/diabetic-foot-ulcer-dfu. Additional data used and analyzed during this study can be obtained from the corresponding author upon reasonable request.
